# Gene expression barcode values reveal a potential link between Parkinson’s disease and gastric cancer

**DOI:** 10.18632/aging.202623

**Published:** 2021-02-16

**Authors:** Suyan Tian, Shishun Zhao, Mingbo Tang, Chi Wang

**Affiliations:** 1 Division of Clinical Research, First Hospital of Jilin University, Changchun 130021, Jilin, P.R. China; 2 Center for Applied Statistical Research, School of Mathematics, Jilin University, Changchun 130012, Jilin, P.R. China; 3 Department of Thoracic Surgery I, First Hospital of Jilin University, Changchun 130021, Jilin, P.R. China; 4 Department of Internal Medicine, College of Medicine, University of Kentucky, Lexington , KY 40536, USA; 5 Markey Cancer Center, University of Kentucky, Lexington, KY 40536, USA

**Keywords:** barcode values, differentially expressed genes (DEGs), gastric cancer, gene expression profiles, Parkinson's disease

## Abstract

Gastric cancer is a disease that develops from the lining of the stomach, whereas Parkinson’s disease is a long-term degenerative disorder of the central nervous system that mainly affects the motor system. Although these two diseases seem to be distinct from each other, increasing evidence suggests that they might be linked. To explore the linkage between these two diseases, differentially expressed genes between the diseased people and their normal controls were identified using the barcode algorithm. This algorithm transforms actual gene expression values into barcode values comprised of 1’s (expressed genes) and 0’s (silenced genes). Once the overlapped differentially expressed genes were identified, their biological relevance was investigated. Thus, using the gene expression profiles and bioinformatics methods, we demonstrate that Parkinson’s disease and gastric cancer are indeed linked. This research may serve as a pilot study, and it will stimulate more research to investigate the relationship between gastric cancer and Parkinson’s disease from the perspective of gene profiles and their functions.

## INTRODUCTION

Gastric cancer (GC), also known as stomach cancer, develops from the cells lining of the stomach. In 2018, more than one million new cases of gastric cancer were diagnosed, and an estimated 783,000 associated deaths occurred. One out of 12 cancer deaths worldwide resulted from GC, making it the fifth most common cancer and the third leading cause of death due to cancer [1]. Gastric cancer poses a particularly serious health problem in Eastern Asia. Parkinson’s disease is a long-term degenerative disorder of the central nervous system that mainly affects the motor system. It is currently listed as the second most prevalent neurodegenerative disorder after Alzheimer’s disease and the most common disorder affecting body movements [2]. The causes of Parkinson’s disease and gastric cancer are very complicated, and increasing evidence supports the involvement of both genetic and environmental factors.

It is believed that the gastrointestinal tract comprises an intrinsic nervous system — the enteric nervous system (ENS). Referred to as “the second brain” [3], the ENS regulates the gastrointestinal tract’s motility and owns neuroendocrine functions. The ENS interacts bi-directionally with the Central Nervous Systems (CNS), in a connection referred to as the “brain-gut axis” [4], which is composed of neural pathways in the CNS, autonomic nervous system, and the hypothalamic-pituitary-adrenal axis. A large number of patients with Parkinson’s disease experience constipation, abdominal distension, and other gastrointestinal symptoms before they experience motor symptoms [5]. More importantly, dysbiosis of gut microbiota plays a critical role in the pathogenesis of Parkinson’s disease, such as [6]. On the other hand, gastrointestinal symptoms and microbiome dysbiosis frequently occur in patients with gastric cancer [7]. Especially, increased gut permeability promotes the leakage of bacteria and their products into the blood, leading to the maturation of antigen-presenting cells and thus the stimulation of inflammatory pathways that are of crucial importance in these two diseases.

Epidemiological studies [8–12] have suggested that patients with Parkinson’s disease have a reduced risk of developing cancers, including gastric cancer, compared to people who do not have Parkinson’s disease. Nevertheless, other studies have showed a positive association between Parkinson’s disease and cancers, that is, an increased risk of cancers in patient with Parkinson’s disease [13, 14]. For instance, for gastric cancer, the hazard ratio was 1.59 (95% CIs: 1.30-1.94) by Lin et al. [14], who attributed the inconsistency to the fact that most of those epidemiological studies were carried out upon the Western population, while their study was performed in Taiwan. Therefore, Lin et al. [14] concluded that the race or/and environmental exposures have an interactive effect on the association between cancers and Parkinson’s disease. Also, the Columbia Open Health Data (COHD) [15], which is based on electronic health records (EHR), indicated that the concurrence of Parkinson’s disease and gastric cancer is significantly higher than what expected by chance (odds ratio=2.02, p=2.23×10^-6^). This implies that both diseases are positively related. It is worth noting that patients in the electronic health records may not represent the general population; thus, the association between the two diseases may be biased. Therefore, it is natural to speculate that Parkinson’s disease and gastric cancer may be linked.

At the molecular level, studies suggest that neurodegenerative disorders (including Alzheimer’s disease, Parkinson’s disease, and Huntington’s disease) and cancers (including lung cancer, liver cancer, and breast cancer) are linked to each other with respect to somatic mutations, mRNAs or microRNAs, such as [16–19]. However, only a few of the studies focused on the specific association between Parkinson’s disease and gastric cancer from this perspective [20, 21]. For example, Hu and colleagues [20] demonstrated that a specific miRNA, miR-148a, is not only a potential tumor suppressor that inhibits gastric cancer metastasis, but is also involved in neurological development and functions. In particular, the expression level of miR-148a is lower in patients with Parkinson’s disease compared to that in normal controls.

Microarray and RNA-sequence techniques enable monitoring expression changes of thousands of genes simultaneously. For both gastric cancer and Parkinson’s disease, numerous microarray and RNA-Sequence experiments such as [22–32] have been conducted to distinguish between the diseased patients and normal controls, or predict the progression of the two diseases, with the aid of a variety of bioinformatics tools and statistical methods. To the best of our knowledge, no investigation has been carried out to explore the link between gastric cancer and Parkinson’s disease, using the gene expression profiles generated through either microarray or RNA-Sequencing. The objective of this study is to bridge this gap by using gene expression profiles and the barcode algorithm [33] to investigate the potential association between the two diseases.

## RESULTS

### Differentially expressed genes

**Figure 1:**
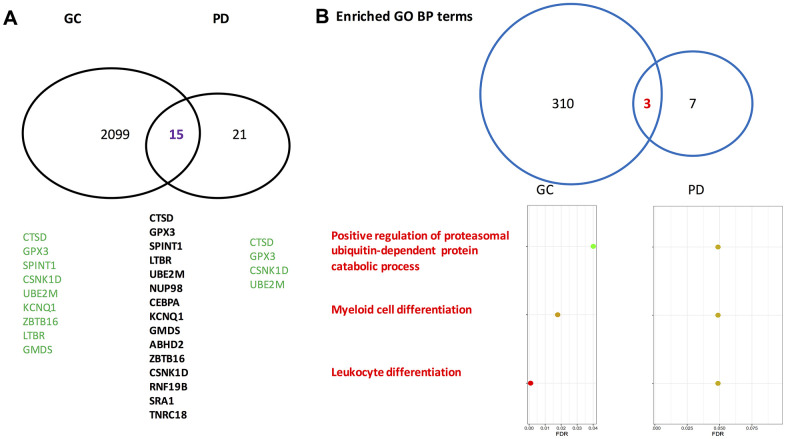
**Venn-diagrams for differentially expressed genes identified by gastric cancer cohort and Parkinson’s disease cohort.** (**A**) On the gene level. (**B**) On the level of enriched Gene Ontology annotation. PD, Parkinson’s disease; GC, gastric cancer; GO, gene ontology; BP, biological process. The gene symbols indicated by the GeneCards database to directly associate with gastric cancer and Parkinson’s disease are highlighted in green. Of note, on the gene level the overlapped rate of gastric cancer and Parkinson’s disease is significant according to a Fisher’s exact test (p=0.033).

For gastric cancer, 2,114 differentially expressed genes (DEGs) between the patients and the normal controls were identified. Among them, 1,296 exhibited a higher expressed proportion, and 818 had a higher silenced proportion higher in the gastric cancer patients compared to the normal controls. For Parkinson’s disease, 36 DEGs were identified. Of them, 33 genes had the propensity of being expressed higher in the disease group than that in the control group. Between the two sets of DEGs, 15 genes overlapped on each other (Fisher’s exact test: p=0.033). The gene symbols for the 15 overlapping genes are presented in Figure 1A, and the odds ratios (ORs) and false discovery rates (FDR) stratified by the gastric cancer cohort and the Parkinson’s disease cohort are listed in Table 1. Of note, in gastric cancer, several overlapped genes had extreme ORs (either 0 or infinite), while in Parkinson’s disease the ORs were basically moderate.

**Table 1:** Overlapped differentially expressed genes by gastric cancer and Parkinson’s disease cohorts.

	**Gastric cancer**		**Parkinson’s disease**	
	OR	FDR	OR	FDR
CTSD	13.073	4.43×10^-23^	2.439	0.010
GPX3	0	1.70×10^-26^	2.320	0.014
SPINT1	4.134	3.27×10^-6^	2.563	0.024
LTBR	11.191	3.40×10^-14^	2.416	0.006
UBE2M	2.719	5.10×10^-4^	4.163	0.030
NUP98	∞	0.013	2.126	0.033
CEBPA	17.688	1.73×10^-8^	2.519	0.005
KCNQ1	0.304	2.30×10^-6^	2.139	0.030
GMDS	3.267	2.16×10^-3^	3.011	0.048
ABHD2	∞	2.07×10^-5^	2.631	0.026
ZBTB16	0.046	3.51×10^-14^	2.116	0.030
CSNK1D	56.713	1.89×10^-14^	2.308	0.034
RNF19B	∞	3.42×10^-5^	2.437	0.010
SRA1	32.759	5.97×10^-9^	2.437	0.013
90-=	2.350	0.025	2.443	0.044

OR: odds ratio, FDR: false discovery rate; ∞: infinite value resulting from have 0’s values in off-diagonal corresponding 2×2 tables.

All genes except *GPX3, ZBTB16,* and *KCNQ1* have OR of >1 for gastric cancer, whereas all genes have OR of >1 for Parkinson’s disease, suggesting that for a patient who has either Parkinson’s disease or gastric cancer, the status of 12 genes is highly likely to be un-silenced. This might imply that when a person suffers from one disease, the likelihood of having the other disease tends to increase, which is consistent with the results of a previous epidemiology study conducted in Taiwan [14] and the high concurrent rate between these two diseases indicated by the COHD database [15]. Nevertheless, Lin’s study [14] indicated that race might play an interactive role on the association between Parkinson’s disease and cancers. Thus, for the Asians, this association tends to positive, but for the Caucasians, this association is more likely to be negative. Since no large Western gastric cancer cohort or Asian Parkinson’s disease cohort on the same microarray platform is available on the GEO database, thus, whether the statement is true cannot be verified using the proposed procedure in this study. Further investigation is warranted. In contrast, the COHD database is based on electronic health records, which may introduce biases to the estimation of the concurrent rate of the two diseases.

**Figure 2:**
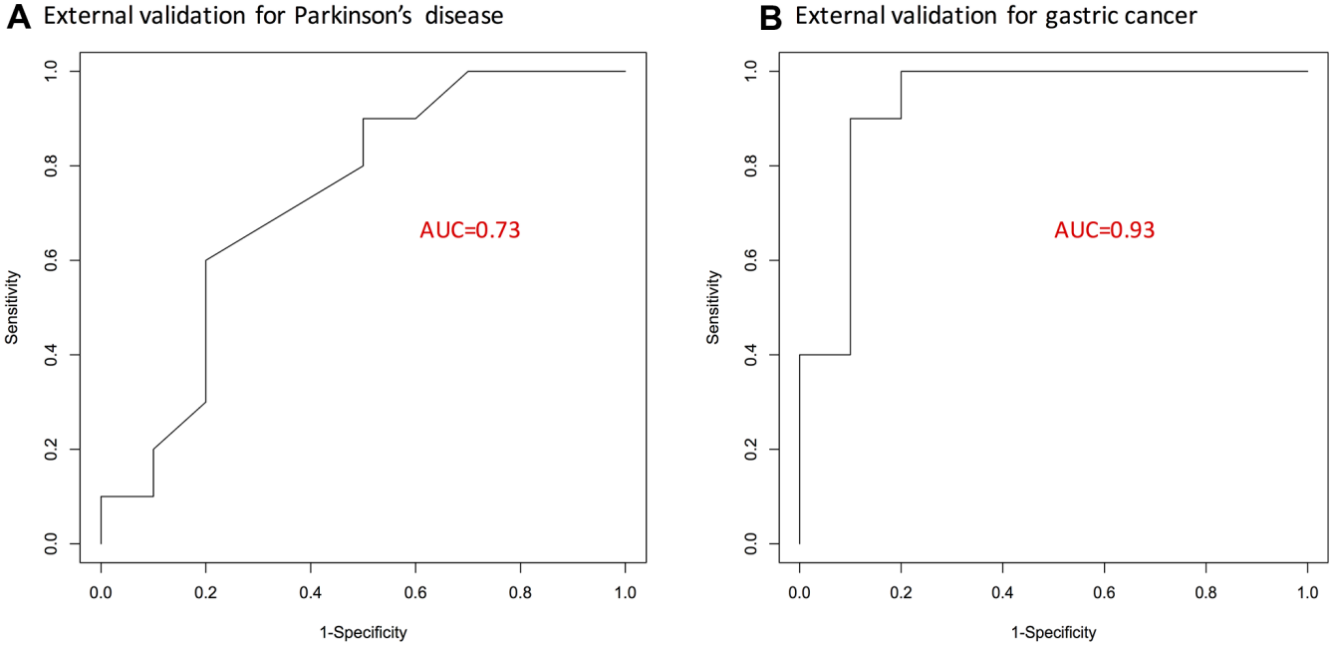
**ROC curves showing predictive performance of the identified 15-gene signature.** (**A**) For Parkinson’s disease. (**B**) For gastric cancer. Here, external validation sets were used. AUC, area under curve; GC, gastric cancer; PD, Parkinson’s disease.

Lastly, a respective logistic regression model with 15 overlapped genes as predictors was fit for either gastric cancer or Parkinson’s disease microarray dataset. The predictive capacity of resulting 15-gene signatures for gastric cancer and Parkinson’s disease was validated on external datasets. As shown by the ROC curves in Figure 2, the list of 15-genes was validated to perform fairly well; especially for gastric cancer, it achieved an AUC statistic of 0.93.

### Pathway enrichment analysis

In the GO biological process category, 313 terms were enriched by the gastric cancer DEGs, and 10 terms were enriched by the Parkinson’s disease DEGs. Among them, three enriched biological process terms were commonly shared by the Parkinson’s disease DEGs and the gastric cancer DEGs. Fifty-one GO chemical component terms were indicated to be enriched by the gastric cancer DEGs, none was enriched by the Parkinson’s disease DEGs. In terms of GO molecular function and KEGG pathway, the numbers are 7 and 17 for gastric cancer DEGs, 0 and 0 for Parkinson’s disease DEGs, respectively. Therefore, no overlapped pathways were found between gastric cancer and Parkinson’s disease regarding the GO molecular function terms, the GO chemical component terms, or KEGG pathways. In Figure 1B, the overlapped GO biological process terms along with their corresponding false discovery rates are presented. Many review articles indicated that both cell proliferation and differentiation as well as ubiquitin-proteasome system play critical roles in the two diseases [34–37].

The three overlapped GO biological process terms deserve further investigation, which may facilitate deciphering the association between Parkinson’s disease and gastric cancer at the gene set level, where the involved genes work in coordination to influence a phenotype of interest.

### Analysis at the network level

**Figure 3:**
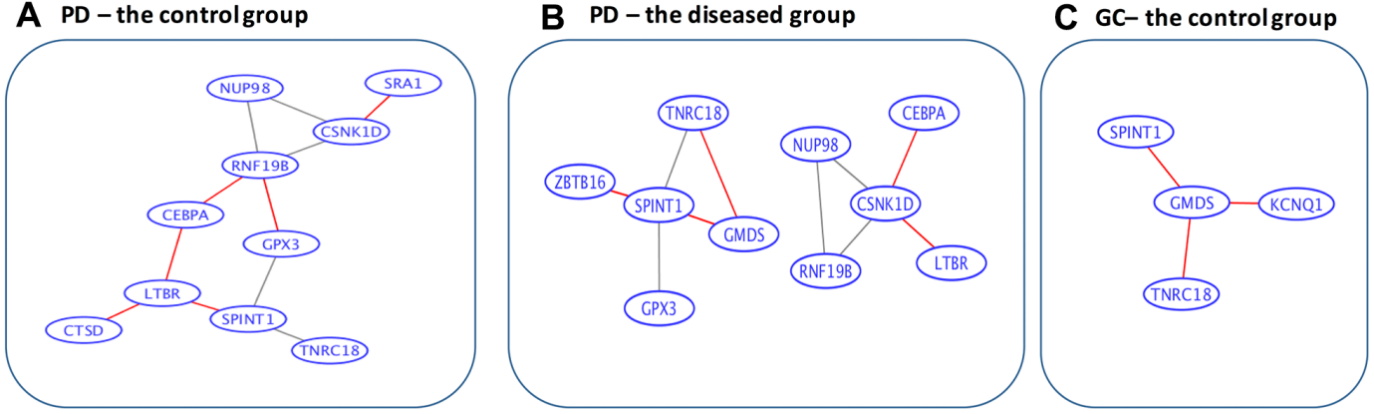
**Data-driven gene-to-gene interaction networks of the overlapped 15 genes.** (**A**) For the control group in Parkinson’s disease. (**B**) For the diseased group in Parkinson’s disease. (**C**) For the control group in gastric cancer. PD, Parkinson’s disease; GC, gastric cancer. The edges highlighted in red are unique for the corresponding categories. Specifically, among the gastric cancer patients, all the 15 overlapped genes are isolated from each other, whereas among the controls, 3 gene pairs (SPINT1 and GMDS, GMDS and TNRC18, and CSNK1D and RNF19B) are connected. In contrast, for Parkinson’s disease patients 5 pairs of connections are gained, 6 pairs are lost, while 5 pairs remain connected.

Using the String software, only four connections (*CSNK1D* to ZBTB16, ZBTB16 to RNF19B, RNF19B to *CSNK1D*, and ZBTB16 to CEBPA) were revealed for the 15 overlapped genes. As a result, a data-driven strategy was used to obtain the information on gene-to-gene interactions (as stated in the Methods section). The corresponding networks for the information on co-expression of the 15 overlapped genes between Parkinson’s disease and gastric cancer (through the calculation of Spearman’s correlation coefficients), are presented in Figure 3. Among the gastric cancer patients, all the 15 overlapping genes appear to be isolated from each other, whereas among the controls, three gene pairs (SPINT1 and GMDS, GMDS and TNRC18, and *CSNK1D* and RNF19B) are connected. This may correspond to a loss of connectivity in the gastric cancer patients, which is consistent with the findings by Anglani et al. [38]. In contrast, for Parkinson’s disease patients, five pairs of connections were gained, six pairs were lost, while five pairs remained connected. Interestingly, two loss-of-connectivity pairs for gastric cancer (SPINT1 and GMDS, and GMDS and TNRC18) were observed among the five gain-of-connectivity pairs for Parkinson’s disease, implying a possible opposite direction of change at the gene-to-gene interaction level for Parkinson’s disease and gastric cancer.

## DISCUSSION

### Biological relevance

The biological relevance of the 15 overlapping genes to gastric cancer and Parkinson’s disease was explored in the GeneCards database [39], and PubMed was searched for more recent information. The GeneCards [39] search revealed that nine of the 15 overlapping DEGs are directly associated with gastric cancer. Meanwhile, four genes related to Parkinson’s disease (*CTSD, GPX3, CSNK1D*, and *UBE2M*) are included in the nine genes directly related to gastric cancer. The remaining overlapping genes are all indirectly associated with either gastric cancer or Parkinson’s disease.

According to the GeneCards [39], Cathespin D (CTSD) exhibits pepsin-like activity and plays a role in protein turnover and in the proteolytic activation of hormones and growth factors. CTSD may be involved in the pathogenesis of several diseases, including breast cancer and Alzheimer's disease. The pathways related to CTSD include lysosome and degradation of the extracellular matrix, and related GO annotations include aspartic-type endopeptidase activity. Liu et al. [40] showed through western blot assay that the CTSD protein is significantly up-regulated in the gastric cancer tissues compared to the normal tissues. Another study [41] showed this regulation pattern using immunohistochemistry. On the other hand, the CTSD levels in Parkinson’s disease patients show a consistent over-expression pattern across several studies, as pointed out in a recent review [42].

The pathways, with which glutathione peroxidase 3 (GPX3) has been associated, include folate metabolism and detoxification of reactive oxygen species, and GO terms are transcription factor binding and selenium binding. In a recent study [43], using two microarray data, viz, GSE99039 (the dataset we used in this study) and GSE72267 as the training set, GPX3 was identified as a DEG for Parkinson’s disease as well. Subsequently, the over-expression of this gene in the diseased tissues was experimentally validated by qRT-PCR. In contrast, GPX3 expression was shown to be lower in gastric cancer patients compared to the normal tissues, and the overexpression of GPX3 can inhibit gastric cancer cell migration and invasion [43]. Meanwhile, another recent study [44] used the The Cancer Genomic Atlas data to show that GPX3 was hypermethylated in gastric cancer, which may consequently increase the possibility of tumor recurrence.

The related pathways of casein kinase 1 delta (CSNK1D) are neuroscience and organelle biogenesis and maintenance. GO annotations related to this gene include transferase activity, transferring phosphorus-containing groups and protein tyrosine kinase activity. UBE2M gene codes for Ubiquitin conjugating enzyme E2 M protein. Among its related pathways are signaling by GPCR and regulation of activated PAK-2p34 by proteasome mediated degradation. GO annotations related to this gene include ubiquitin-protein transferase activity and ubiquitin protein ligase activity. As far as these two genes are considered, there are no recent experiments reported in PubMed to provide more support on their relevance to either Parkinson’s disease or gastric cancer.

Of note, a long non-coding RNA, steroid receptor RNA activator 1 (*SRA1*), has been experimentally validated to play roles in a variety of cancer types, including breast cancer, prostate cancer, and liver cancer. While there is no experimental evidence on its association with gastric cancer and Parkinson’s disease, it was predicted to associate with these two diseases using computational methods in the lncRNADisease2.0 database [45]. Therefore, exploration of the potential association between these two diseases in terms of lncRNAs may be a promising research avenue.

## CONCLUSIONS

Using gene expression profiles and the barcode algorithm, we show that two distinct diseases, Parkinson’s disease, and gastric cancer are indeed linked to each other at the molecular level. Our future work will focus on two questions, whether the association is positive or negative and whether and how race or certain environmental factors influence the association between these two diseases.

To conclude, the present study may serve as a pilot study, and it may inspire more research to evaluate the relationship between cancer and neurodegenerative diseases from the perspective of genes and their interaction networks.

## MATERIALS AND METHODS

### Experimental data

The barcode algorithm was used in this study to identify differentially expression genes between the diseased group and the control group. Therefore, some restrictions on the microarray platforms were imposed. Specifically, for human studies, chips that are applicable to the barcode algorithm include Affymetrix U133A (GPL96), U133 2.0 (GPL571), U133plus 2.0 (GPL570), and human gene 1.0 ST (GPL6244) because a large number of chips are required to estimate the null mean and variance in the method.

To acquire a sufficient statistical power to evaluate the association between these two diseases, the sample sizes of both gastric cancer and Parkinson’s disease cohorts need to be large. As a result, two microarray experiments in the NIH’s Gene Expression Omnibus (GEO) repository from the National Institute of Health were selected: GSE99039 [46] for Parkinson’s disease and GSE66229 [47] for gastric cancer. In addition, GSE20146 [22] and GSE79973 [28] were used as external validation sets to evaluate the predictive performance of the resulting gene list. The demographical characteristics of these four studies are summarized in Table 2.

**Table 2:** Characteristics of microarray experiments in this study.

	**Reference**	**Raw data**	**Platform**	**Diseased**	**Controls**	**Race**
Training set						
GSE99039 (Parkinson’s disease)	[46]	Yes	GPL570	205	233	Western
GSE66229 (gastric cancer)	[47]	Yes	GPL570	303	101	Asian
Validation set						
GSE20146 (Parkinson’s disease)	[22]	Yes	GPL570	10	10	Western
GSE79973 (gastric cancer)	[28]	Yes	GPL570	10	10	Asian

### Pre-processing procedures

Raw data (CEL files) of the two microarray experiments were downloaded from the GEO repository and pre-processed using the fRMA algorithm [48], which can provide effective control on batch effects and enable pre-processing of a single chip [48–50]. For those multiple probe sets matched to the identical gene, the one with the largest absolute log fold change was retained.

### Statistical methods

### Barcode algorithm

The barcode algorithm proposed by McCall et al. [33] transformed the actual expression values into binary barcode values, and the expressed genes are coded with 1’s and the silenced genes are coded with 0’s. Briefly, for each gene, a mixture model in the algorithm is used to fit the silenced and expressed distribution of observed log2 transformed intensity values. The mixture model is expressed as:

$$Y_{ig}|\mu_{g}\sim \left(1-p_{g}\right)\times N\left(\mu_{g},\;\tau_{g}^{2}\right)+p_{g}\times U\left(\mu_{g},S_{g}\right)$$

$$\mu_{g}\sim N\left(\xi ,\;\lambda^{2}\right)$$

$$\tau_{g}^{2}\sim IG\left(\alpha ,\;\beta \right)$$

where Y_ig_ is the log2 expression value for gene *g* in sample *i*, and follows a normal distribution of N (μ_g_, τ_g_^2^) when gene *g* is silenced or has a uniform distribution of U(μ_g_, S_g_) when it is expressed. Here, μ_g_ denotes the mean of silenced genes, and S_g_ denotes the saturation value (i.e., the upper limit of gene expression values). Then, μ_g_ and τ_g_^2^ for gene *g* are assumed to follow normal and inverse gamma distributions, respectively. With a hierarchical model structure, and in particular the introduction of higher-level parameters (α, β, ξ, and λ), more stable estimates of variances for silenced genes are expected because the information across genes is borrowed and shared across genes, leading to a shrinkage of estimates for individual genes toward the overall level.

To determine if a gene is silenced or expressed, the standardized intensity value, (y_ig_ − μ_g_)/τ_g_, was calculated. Upon a pre-determined cutoff value C, the expression barcode for a gene, a vector of 1’s and 0’s is defined as,

$$barcode_{ig}=\{\begin{array}{c} 1\;\;\;\;\Phi \left(-\left(y_{ig}-\mu_{g}\right)/\tau_{g}\right)<C\\ \;\;\;0\;\;\;\;otherwise\;\;\;\;\;\;\;\;\;\;\;\;\;\;\;\;\;\;\;\;\;\;\;\;\;\;\;\;\;\; \end{array}$$

where Φ is the cumulative density function of a standard normal, parameter estimation in this hierarchical model is done using a modified expectation-maximization algorithm (the details of the barcode are available in the supplementary material of a previous study [33]). The barcode algorithm was implemented by the barcode function in the R fRMA package, and the default value of C was used.

### Differentially expressed genes

On the barcode values, the genes with all values of either 1’s or 0’s for the respective gastric cancer and Parkinson’s disease cohorts were eliminated, and finally, 8,392 probe-sets were fed into the downstream analysis.

**Figure 4:**
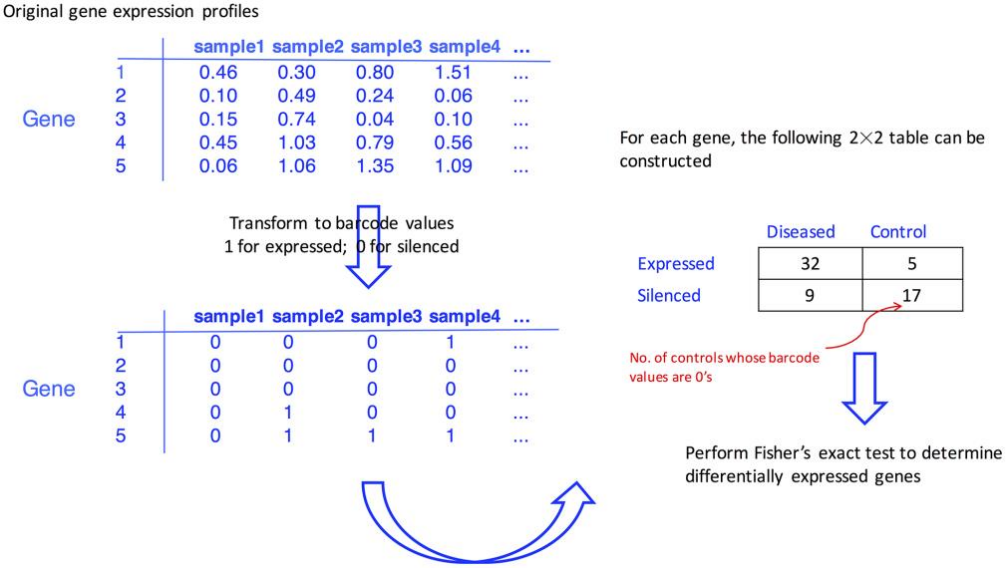
**Flowchart illustrating how the differentially expressed genes are identified with the aid of barcode algorithm.**

To determine if the expressed ratios differed in the diseased group versus the control group, Fisher’s exact test for individual genes was carried out upon the barcode values. Genes with a false discovery rate (FDR) of < 0.05, which was calculated through the Benjamini-Hochberg (BH) procedure [51] to adjust for multiple testing issue, were considered as differentially expressed genes in the respective gastric cancer and Parkinson’s disease cohorts. The flowchart of the proposed procedure is given in Figure 4.

### Pathway enrichment analysis and network construction

Using the R clusterProfilter package, the Kyoto Encyclopedia of Genes and Genomes (KEGG) pathway enrichment analysis and Gene Ontology (GO) functional annotation were carried out on the differentially expressed genes of the gastric cancer cohort and the Parkinson’s disease cohort, respectively. In these analyses, all default parameters were used, and minimum gene set size parameter was set at 5 in the enrichGO function and the enrichKEGG function.

Information on data-driven gene-to-gene interaction information was obtained by calculating Spearman’s correlation coefficients among the overlapped differentially expressed genes. If the absolute value of Spearman’s correlation coefficients is >0.4 and the corresponding false discovery rate is < 0.05, the specific gene pair is connected. Otherwise, they are not connected. The resulting data-driven gene-to-gene interaction information was used to plot network graph in the Cytoscape software [52].

### Biological relevance

The GeneCards database [39] was mined to investigate the biological relevance of identified differentially expressed genes for gastric cancer and Parkinson’s disease. In addition, PubMed was searched for more recent literature on the potential relationship between the overlapped differentially expressed genes with gastric cancer and Parkinson’s disease.

### Statistical language

All statistical analyses were carried out in R 3.3 (https://www.r-project.org/).

### Availability of data and materials

Four microarray datasets were downloaded from the GEO database, the data are open and publicly available.
